# Internet addiction and excessive daytime sleepiness in adolescents

**DOI:** 10.1192/j.eurpsy.2021.1546

**Published:** 2021-08-13

**Authors:** N. Semenova, S. Tereshchenko, L. Evert L, O. Zaytseva, M. Shubina

**Affiliations:** Scientific Research Institute For Medical Problems Of The North, Federal Budgetary Scientific Institution «Federal Research Centre «Krasnoyarsk Scientific Centre of Siberian Division of Russian Academy of Sciences», Krasnoyarsk, Russian Federation

**Keywords:** Internet, Addiction, sleep disorders

## Abstract

**Introduction:**

Internet addiction (IA) is associated with personal peculiarities, psychosocial characteristics, and physiological factors. Excessive daytime sleepiness (EDS) is one of the most common sleep disorders in adolescents associated with social behavior patterns.

**Objectives:**

To evaluate the association of IA with EDS in Siberian adolescents.

**Methods:**

4637 urban Siberian (Krasnoyarsk, Abakan) school-based adolescents (aged 12-18; boys/girl ratio 2215/2422) were tested with Chen Internet Addiction Scale (CIAS). Internet users were categorized into three groups: adaptive Internet users (AIU) (scoring 27–42); maladaptive Internet users (MIU) (scoring 43–64); and pathological Internet users (PIU) (scoring ≥65). EDS was assessed by Pediatric Daytime Sleepiness Scale (PDSS); cutoffs for EDS were PDSS 95% percentiles for each age group: 12 y.o. – 20 points, 13 y.o. – 21 points, 14-16 y.o. – 22 points, 17-18 y.o. – 23 points. Quantitative data are shown as median (25-75% quartiles). Chi-square and Kruskal-Wallis tests were used.

**Results:**

EDS prevalence increase with IA severity: AIU group (n=2402) – 1.4%, MIU group (n=1905) – 3%, and PIU group (n=330) – 12.4% (pAIU-MIU=0.001; pAIU-PIU<0.001; pMIU-PIU<0.001). Positive association was detected between IA severity and PDSS score: AIU group – 10 (6-13), MIU group – 14 (10-17), and PIU group – 17 (13-21), p(K-W)<0.001).

**Conclusions:**

EDS is associated with IA in Siberian adolescents. The possible explanations of this relation may be: (1) the higher rate of night activity, (2) night sleep disturbances and (3) the presence of common pathogenic factors in IA and EDS, such as personality characteristics, depression, anxiety. The study was funded by RFBR project № 18-29-2203219.

**Conflict of interest:**

The reported study was funded by RFBR according to the research project № 18-29-22032\19.

